# Parry-Romberg Syndrome With Localized Scleroderma: A Report of Two Pediatric Cases From Oman

**DOI:** 10.7759/cureus.95619

**Published:** 2025-10-28

**Authors:** Manhal H Al Lawati, Wafaa Al Shehhi, Fatema Al Amrani, Ruqaiya Al Jashmi, Amna Al-Futaisi

**Affiliations:** 1 Medicine, Sultan Qaboos University, Muscat, OMN; 2 Neurology, The Royal Hospital, Muscat, OMN; 3 Pediatric Neurology, Sultan Qaboos University, Muscat, OMN; 4 Pediatric Rheumatology, The Royal Hospital, Muscat, OMN; 5 Pediatrics, Sultan Qaboos University, Muscat, OMN

**Keywords:** autoimmune diseases, en coup de sabre, facial atrophy, localized scleroderma, parry-romberg syndrome, pediatric dermatology, progressive hemifacial atrophy

## Abstract

Parry-Romberg syndrome (PRS) is a rare, acquired neurocutaneous disorder characterized by progressive hemifacial atrophy, primarily affecting the skin, soft tissue, and underlying bone. The etiology remains unclear, though autoimmune, inflammatory, and genetic factors have been implicated. PRS frequently coexists with localized scleroderma (en coup de sabre), leading to diagnostic and therapeutic challenges. Here, we present two pediatric cases from tertiary care hospitals in Oman, emphasizing clinical presentation, diagnostic findings, and treatment response.

The first patient, a 10-year-old male, presented with progressive left-sided facial atrophy since age seven, associated with green discoloration beneath the left eye. Skin biopsy confirmed epidermal atrophy with lipodystrophy. MRI revealed asymmetric lateral ventricles with mild left facial atrophy. He was treated with IV methylprednisolone, followed by oral prednisolone and methotrexate, resulting in improved soft tissue bulk.

The second patient, a nine-year-old female, developed right-sided facial atrophy at age five, with a hyperpigmented macule on the mandible. MRI was normal, while histopathology showed epidermal atrophy with increased basal pigmentation and dermal fibrosis. She initially received methotrexate but developed transaminitis, necessitating a switch to mycophenolate mofetil. Disease progression was halted following immunosuppressive therapy.

PRS predominantly affects females and typically presents in the first two decades of life. Our cases align with reported literature, with both patients developing symptoms in early childhood. Neurological involvement, commonly reported in PRS, was absent in our patients. MRI findings were inconsistent with the literature, as one patient exhibited mild atrophy while the other had a normal scan. The presence of dental anomalies, including malocclusion and caries, underscores the multisystemic impact of PRS. Current treatment strategies focus on halting progression using corticosteroids and immunosuppressants, as demonstrated by favorable outcomes in our cases.

PRS remains a diagnostic challenge due to its variable presentation and uncertain pathogenesis. Early recognition and immunosuppressive therapy can mitigate disease progression and improve outcomes. Further studies are needed to elucidate long-term prognostic factors and optimal management strategies.

## Introduction

Progressive hemifacial atrophy, or Parry-Romberg syndrome (PRS), is a rare acquired idiopathic neurocutaneous disorder [1]. It is characterized by slowly progressive craniofacial asymmetry due to atrophy affecting one side of the face that is self-limiting in nature [2]. This syndrome was first described in the 19th century by Caleb Parry and Moritz Romberg [3].

It typically presents in the pediatric age group (below 20 years), with a female predominance, as reports have shown that females are three times more likely to develop the condition when compared to males [1,3-5]. However, there are several case reports with later presentation, beyond the age of 20 years.

It was noticed that PRS is associated with linear scleroderma, known as "en coup de sabre" (42%) [2], a localized form of morphea, commonly occurring in the scalp (frontoparietal) and forehead. Some reports describe the lesions as typically starting in the maxillary and periorbital region and later advancing to the rest of the face on the affected side [4]. A few reports have mentioned bilateral involvement as well [3].

The exact pathophysiology of this condition is yet to be fully understood [4]. However, some theories suspect that fat metabolism disturbances, viral infections, inborn errors of immunity, and inheritance may be potential factors directing the pathogenesis of PRS [3].

Fifteen to twenty percent of patients with PRS present with neurological manifestations, most commonly ipsilateral headaches [4], atypical migraines, or trigeminal neuralgia, which are seen in more than half of the patients with neurologic symptoms (52%). Other symptoms include facial pain, seizures, and hemimasticatory spasms [2].

A diagnosis of PRS can be established clinically when progressive atrophy is described, presenting as a sunken cheek, eye, and tongue. Blood investigations would show high anti-nuclear antibody titers, especially with active linear scleroderma [3]. If the patient has neurological complaints, computed tomography (CT) and magnetic resonance imaging (MRI) should be performed [3], which may show hyperintense ipsilateral cortical and subcortical lesions, intraparenchymal calcifications, and localized cerebral hemiatrophy, corresponding to the affected side. EEG recording can also be performed to look for continuous slowing and multifocal sharp waves [2]. A biopsy of the skin lesions is recommended for histopathological assessment of localized scleroderma.

PRS as a separate entity does not usually require treatment, but the coexistence of localized scleroderma or other underlying inborn errors of immunity necessitates the use of methotrexate and corticosteroids in most cases [6]. If the condition requires more aggressive therapy, other immunosuppressants such as cyclophosphamide and cyclosporine are considered [2].

Here, we present two pediatric patients who presented with progressive unilateral facial atrophy and were found to have localized scleroderma lesions as well, from two tertiary care hospitals in Oman.

## Case presentation

Here we describe two pediatric cases diagnosed with PRS at tertiary care hospitals in Oman. Both patients presented with progressive unilateral facial atrophy and were evaluated for associated neurological and dermatological manifestations. A detailed comparison of their clinical features, diagnostic findings and treatment outcomes is provided in Table 1.

**Table 1:** Clinical and Diagnostic Findings of Two Pediatric Cases of Parry-Romberg Syndrome in Oman AB-PAS: Alcian Blue-Periodic Acid-Schiff; DMARD: disease-modifying antirheumatic drug; MTX: methotrexate

	Sultan Qaboos University Hospital	Royal Hospital
Current age of the patient	10 years	9 years
Age at presentation	7 years	5 years
Age at diagnosis	9 years	8 years
Gender	Male	Female
Presence of facial discoloration/linear scleroderma	Green discoloration beneath the left eye.	A hyperpigmented macule with an irregular border on the right side of the mandible.
Skin biopsy findings	Epidermal atrophy with lipodystrophy.	Epidermis with focal loss of rete and increased pigmentation in basal layers. Papillary dermis shows dilated capillaries and focal edema and few melanophages. There is mild perivascular and focal perifollicular lymphocytic exocytosis with vacuolar degeneration. Follicular mesenchyme bodies, atrophic follicular remnants and eccrine glands are noted in upper dermis. Special stain AB-PAS is negative for interstitial mucin.
MRI findings	Brain showed asymmetric lateral ventricles, larger on the left, and mild facial atrophy on the left side but no signal abnormalities.	No abnormality detected.
CT findings	No abnormality detected, no calcifications.	N/A
Maxillo-facial findings/dental anomalies	Malocclusion, no other anomalies.	Dental crowding and caries.
Eye exam findings	Normal ocular examination.	Normal ocular examination.
Other investigations done for the patient	Non-contrast enhanced CT of paranasal sinuses: Small areas of mucosal thickening are seen in both maxillary sinuses, no definite focal bony lesion is seen.	N/A
Treatment received (dose & duration)	IV methylprednisolone, oral prednisolone 10 mg with quick tapering over a month, currently on prednisolone 5 mg OD and MTX 15 mg weekly.	IV methylprednisolone then oral prednisolone 1 mg/kg with slow tapering, Initailly MTX 15 mg/m^2^, then upgraded to mycophenolate mofetil (MMF) 600 mg/m2 twice daily
Outcome	Softening of the skin with improved mass bulk on left cheek.	Progression stopped since she received prednisolone and DMARDs, developed transaminitis secondary to MTX then switched to MMF

Case 1

**Figure 1:**
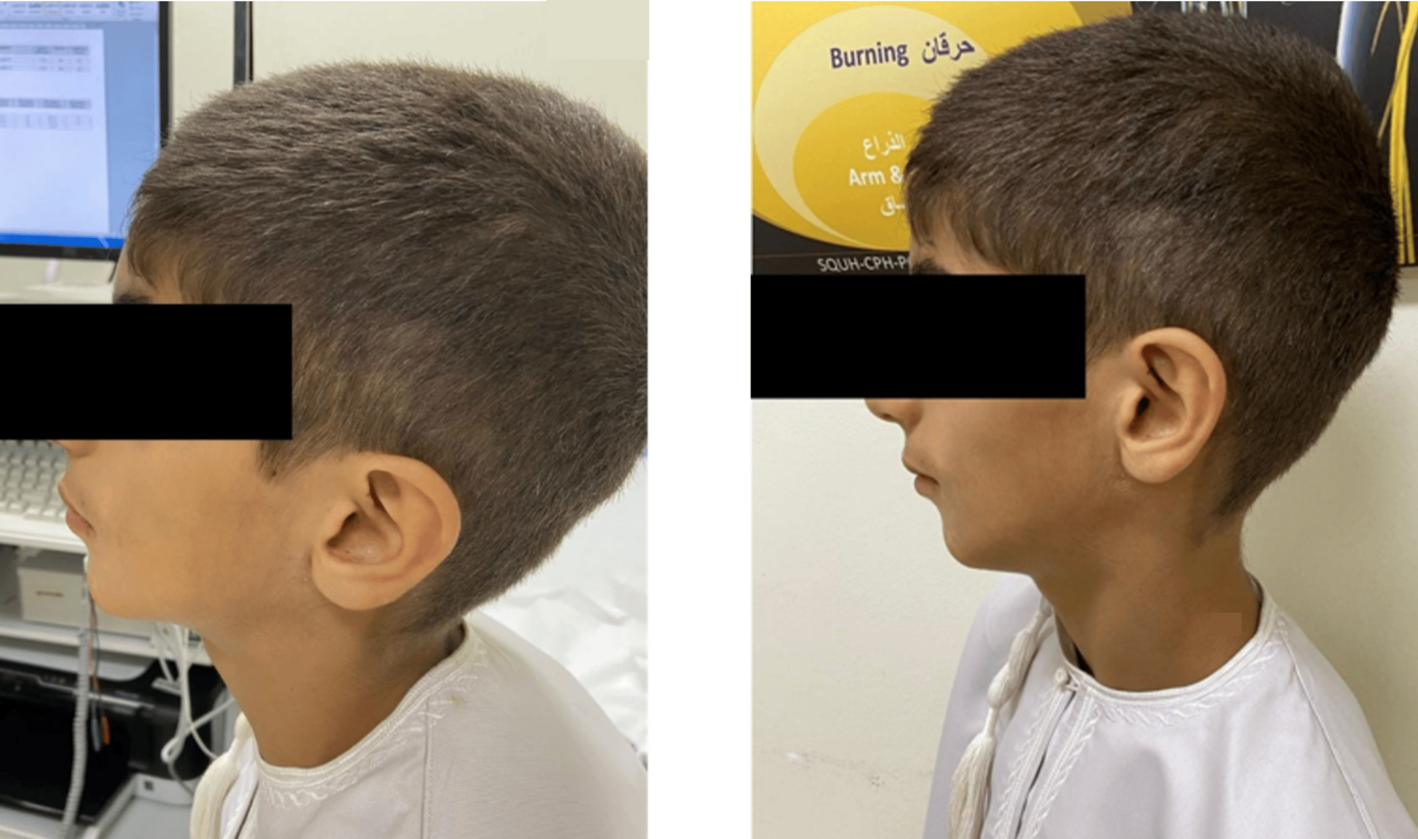
Clinical Presentation of Patient 1 With Left-Sided Hemifacial Atrophy The patient provided informed consent for the publication of their images.

A 10‑year‑old male with an onset of left‑sided facial atrophy at age seven, initially noted by subtle flattening and green discoloration under the left eye. On exam, there was skin thinning, loss of subcutaneous fat and cheek volume on the left side (Figure 1). MRI revealed mild left facial soft‑tissue atrophy with asymmetric lateral ventricles (larger left ventricle) but no signal abnormalities. Skin biopsy showed epidermal atrophy with lipodystrophy. He was treated with intravenous methylprednisolone followed by a tapering course of oral prednisolone (5 mg daily) and weekly methotrexate (15 mg). Following three months of therapy, soft‑tissue bulk improved, and skin texture softened.

Case 2

**Figure 2:**
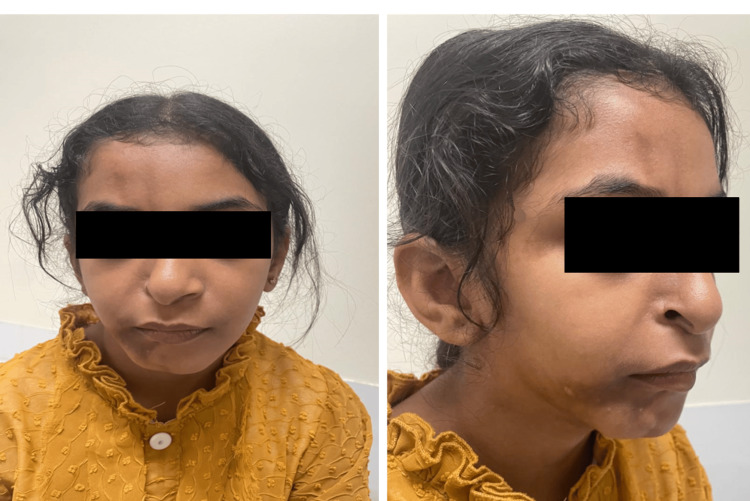
Clinical Presentation of Patient 2 With Right-Sided Hemifacial Atrophy The patient provided informed consent for the publication of their images.

A nine-year‑old female whose right‑sided facial atrophy began at age five, accompanied by a hyperpigmented macule on the mandible (Figure 2). Dental evaluation revealed malocclusion and multiple carious teeth. MRI was normal; histopathology showed epidermal thinning, increased basal pigmentation, dermal fibrosis and mild lymphocytic exocytosis. She initially received methotrexate (15 mg/m²) but developed hepatic transaminitis and was switched to mycophenolate mofetil (600 mg/m² twice daily). Disease progression has been halted, and facial asymmetry remains stable.

## Discussion

PRS is a rare disease entity, with approximately 150 cases reported in the literature [7]. Patients with PRS are typically normal at birth, and symptoms start appearing in the first two decades of life, often around 10 years of age [8,9]. This is consistent with our patients, who presented in their first decade of life [7].

The most commonly affected organs in PRS are the eyes, skin, and nervous system. Headaches, particularly migraines, are widely seen in these patients, along with facial paresthesia, focal epilepsy, and trigeminal neuralgia [9,8,3]. However, neither of our patients exhibited specific neurological abnormalities other than unilateral facial weakness, nor did they have ocular involvement.

The predominant affected organ in our cases was the skin, with both patients showing discoloration on one side of the face, with differences in lesion size, margins, and affected side. The final deformity depends on the disease course; the later it is discovered, the greater the damage [9].

PRS and localized scleroderma have overlapping features, leading some clinicians to suggest they are the same entity. However, no consensus has been established [8]. Diagnosis of PRS is confirmed through skin biopsy, which reveals atrophy of subcutaneous tissue, fat, fascia, and sometimes muscle, cartilage, and bone, with minimal epidermal involvement and fibrosis [10]. Hyperpigmentation or depigmentation, atrophic hair follicles, and alopecia are also common findings [8]. This aligns with the biopsy findings in our Sultan Qaboos University Hospital (SQUH) patient, which showed epidermal atrophy with lipodystrophy.

MRI findings of PRS commonly show asymmetric fatty loss in the frontal area without cortical or white matter involvement [7]. However, this was not prominent in our cases, as MRI findings were minimal or absent. PRS patients also frequently have dental anomalies, including maxillary and mandibular hypoplasia due to bone resorption, leading to crowding of teeth and a higher risk of early dental caries [4,9]. Both of our patients demonstrated crowded teeth with malocclusion and caries.

PRS is considered a self-limiting illness with no definitive cure [8,7]. Corticosteroids are used during the active stage to slow progression, with immunosuppressants such as methotrexate, cyclophosphamide, cyclosporine, and hydroxychloroquine when intensive management is required [2]. In our patients, both steroids and various immunosuppressive agents were used with favorable outcomes.

## Conclusions

PRS is a rare and poorly understood disorder with a variable clinical presentation, making early diagnosis challenging. Its unpredictable progression complicates prognosis, and current treatment focuses on immunosuppression to manage symptoms and slow disease progression. Further research is required to better understand PRS and develop targeted therapies.
